# Comparative Analysis of Cluster of Differentiation 57 and Proliferating Cell Nuclear Antigen Expression in Different Grades of Oral Squamous Cell Carcinoma: An Immunohistochemical Study

**DOI:** 10.7759/cureus.44779

**Published:** 2023-09-06

**Authors:** Ani Simila CS, T Isaac Joseph, KL Girish, Prasanth T, Angelin Binu, Jeslin Mary

**Affiliations:** 1 Oral and Maxillofacial Pathology, Rajas Dental College and Hospital, Kavalkinaru, IND; 2 Oral and Maxillofacial Pathology, Sree Mookambika Institute of Dental Sciences, Kulasekaram, IND

**Keywords:** pcna, oral squamous cell carcinoma, immunohistochemistry, proliferating cell nuclear antigen, cd57

## Abstract

Background: The immune defense against tumor cells is mainly mediated by the natural killer (NK) cells. Cluster of differentiation 57 (CD57) is a 110-kd glycoprotein, typically expressed by the NK cells, attacks the cancer cells and inhibits tumor development. Proliferating cell nuclear antigen (PCNA) is a 36-kd auxiliary protein for DNA polymerase delta that correlates with cell proliferation and DNA synthesis. It is an essential component of DNA replication, DNA recombination, and DNA repair. The uncoordinated proliferation of PCNA protein characterizes the biological behavior of malignant lesions.

Aim: The aim of the present study is to compare and correlate the expression of CD57 and PCNA in different grades of oral squamous cell carcinoma (OSCC) by immunohistochemistry.

Materials and methods: This retrospective analysis comprises 30 samples of various grades of OSCCs and 10 samples of healthy mucosa. Sections of 4-5 µm thickness were done and stained with monoclonal anti-PCNA and anti-CD57 antibodies. The statistical package for social science (SPSS) version 16.0 software (IBM Corp., Armonk, NY) was used to analyze the data in this study. The expression of CD57 and PCNA was compared and correlated between the groups using analysis of variance (ANOVA) post hoc, Dunnet *t-test*, and Pearson's correlation coefficient test. For statistical significance, a p-value of 0.05 or less was used.

Results: A significant decrease in CD57 labeling index was seen from well-differentiated squamous cell carcinoma (16.63 ± 2.33) to poorly differentiated squamous cell carcinoma (5.53 ± 1.20) whereas the significant increase in PCNA labeling index was noted from well-differentiated squamous cell carcinoma (45.88 ± 2.20), followed by moderately differentiated and poorly differentiated squamous cell carcinoma (72.77 ± 4.35).

Conclusion: The combination of CD57 and PCNA biomarkers appears to be good indicators of the immune status of the patient and the aggressiveness of the lesion.

## Introduction

Multicellular organisms can sustain their life only when all the cells function in accordance with the rules that regulate cell growth and reproduction [1]. The cell numbers are maintained by controlling the rate of cell division as well as the death of the cells. Thus, mitosis and apoptosis maintain the normal homeostasis of the body. Dysregulation of these mechanisms may lead to either increase or decrease in the cell number [2].

Normal control systems prevent the tumor cells from undergoing cellular proliferation and differentiation. When these control mechanisms get altered, they undergo cellular proliferation that leads to uncontrolled cell growth even in the presence of the signals that normally inhibit cell growth and division [1]. Due to mutations in the proto-oncogenes, tumor suppressor genes, and DNA repair genes, the cells exhibit uncontrolled proliferation and spread rapidly resulting in invasion and metastasis [1].

Natural killer (NK) cells play an important role in the innate and adaptive immune system. The immune defense against tumor cells is mainly mediated by the NK cells [3]. These cells detect and limit the development of the tumor directly without any priming or prior activation [4]. NK cells identify the tumor cells by the lack of expression of major histocompatibility complex-I and secrete cytokines like interferon-ϒ and tumor necrosis factor-α [3].

A cluster of differentiation 57 was identified on NK cells by using the mouse monoclonal antibodies human natural killer-1 (HNK1) and Leu-7 and it was assigned as a cluster of differentiation (CD) designation, CD57, at the fourth International Workshop of Human Leukocyte Antigens in 1989 [5-6]. CD57 is a 110-kd glycoprotein that is typically expressed by the NK cells, which attack the cancer cells and inhibit tumor development [4].

Proliferating cell nuclear antigen (PCNA - cyclin) is a 36-kd auxiliary protein for DNA polymerase delta, located on chromosome 20p12 which correlates with cell proliferation and DNA synthesis. This PCNA protein varies during the cell cycle and accumulates in the late G and S phases of the cell cycle [7]. This protein is increased in the G1, and S phases and it is decreased in the G2 phase. Hence, this protein is a reliable indicator of cell proliferation [8]. PCNA is an essential component of DNA replication, DNA recombination, and repair. The malignant tissue is also characterized by an uncoordinated proliferation of this antigen namely, PCNA [9].

Since cellular proliferation and the presence of NK cells are important indicators for the aggressive nature and prognosis of the disease, these biomarkers are useful in predicting the aggressiveness of the disease as well as the immune status of the individual [7] and so this study aims to use CD57 and PCNA biomarkers to determine the immune status as well as aggressiveness or the biologic behavior of the disease thereby helping in the planning of the treatment.

## Materials and methods

This study was a retrospective study conducted in the Department of Oral Pathology and Microbiology at Sree Mookambika Institute of Dental Sciences, Kulasekharam for a period of one year. A total of 40 samples were taken from the archival blocks of our department that were reported during the year 2011-2018. The control group (Group I) consisted of 10 blocks of normal mucosa and the study group comprised 30 blocks of different grades of oral squamous cell carcinoma. Tissue sections taken from the paraffin-embedded blocks that are clinically diagnosed and microscopically confirmed cases of well-differentiated squamous cell carcinoma (WDSCC-Group II), moderately differentiated squamous cell carcinoma (MDSCC-Group III), and poorly differentiated squamous cell carcinoma (PDSCC-Group IV) were the inclusion criteria of this study. Each group comprised 10 blocks respectively. Archival blocks in which antigen retrieval was not possible were the exclusion criteria.

Methodology

**Figure 1:**
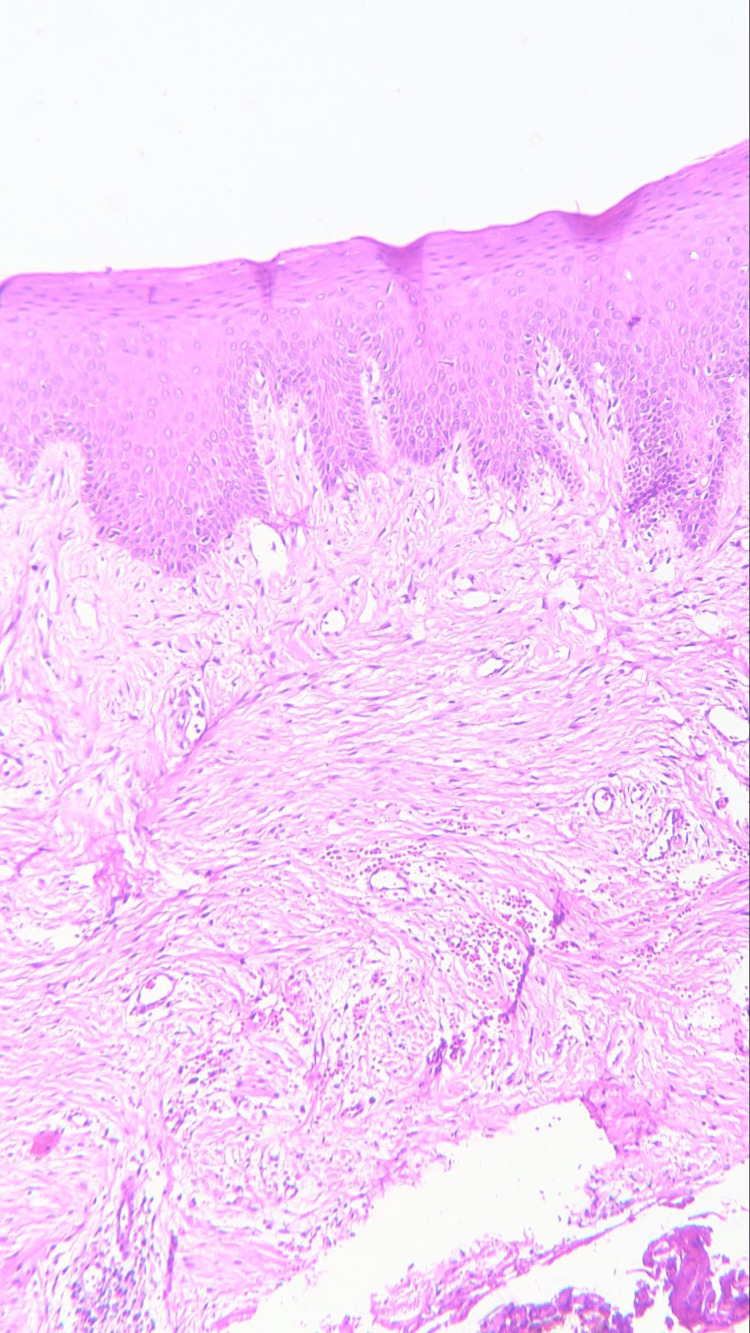
Photomicrograph showing histology of normal mucosa (H&E staining; 100x).

**Figure 2:**
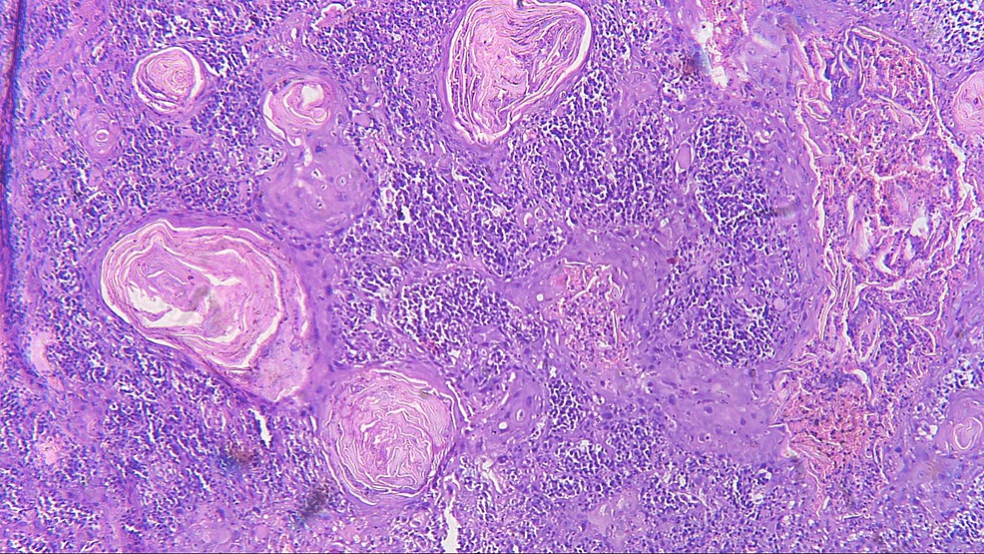
Photomicrograph showing histopathology of WDSCC (H&E staining; 100x). WDSCC, well-differentiated squamous cell carcinoma

**Figure 3:**
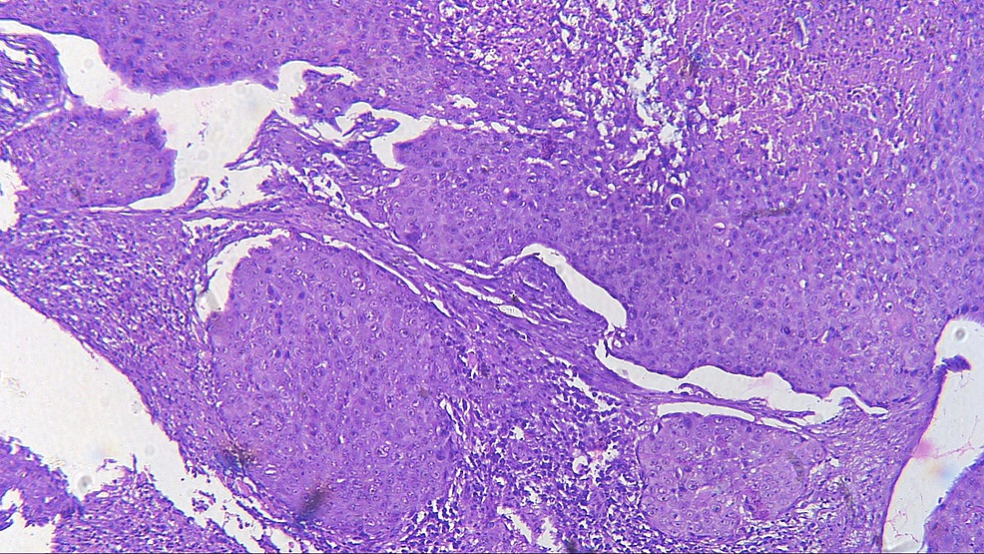
Photomicrograph showing histopathology of MDSCC (H&E staining; 100x). MDSCC, moderately differentiated squamous cell carcinoma

**Figure 4:**
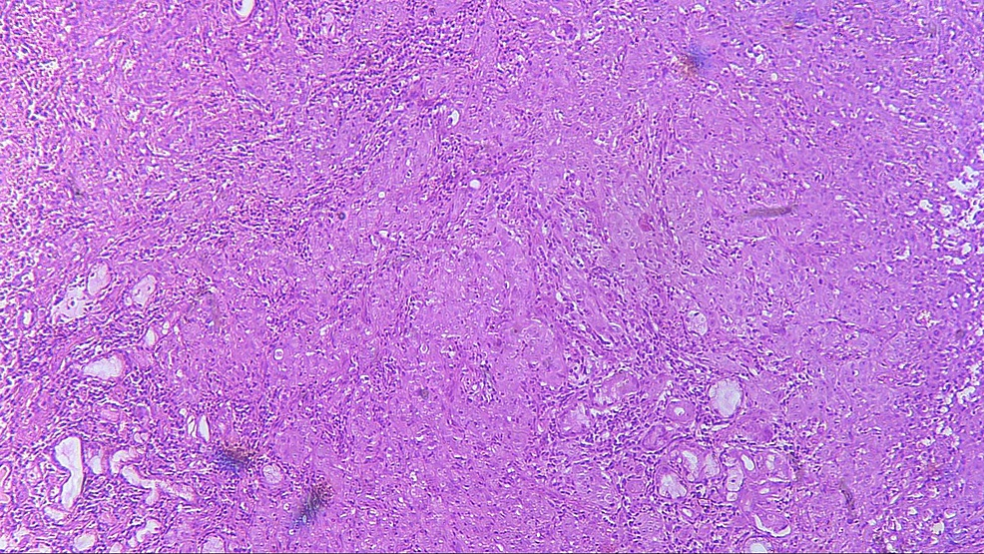
Photomicrograph showing histopathology of PDSCC (H&E staining; 100x). PDSCC, poorly differentiated squamous cell carcinoma

**Figure 5:**
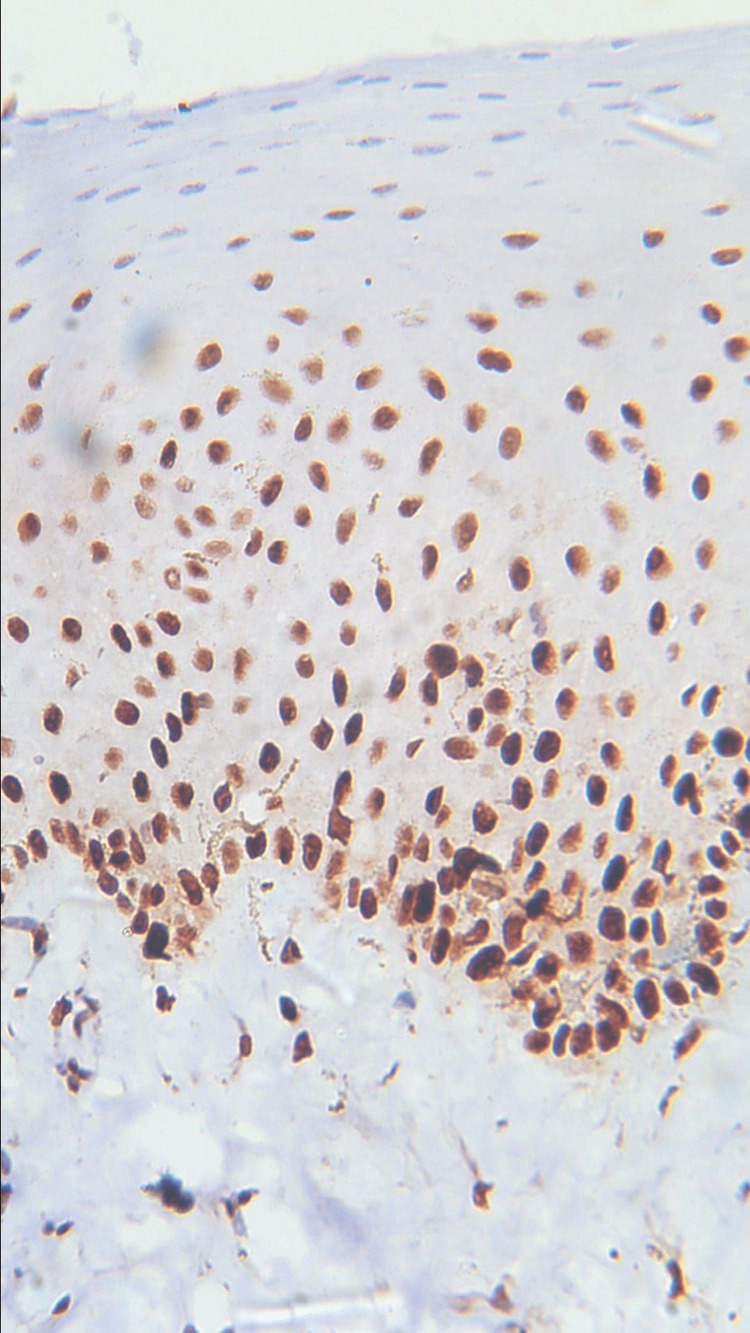
Photomicrograph showing expression of PCNA in normal mucosa (IHC staining; 400x). IHC, immunohistochemistry; PCNA, proliferating cell nuclear antigen

**Figure 6:**
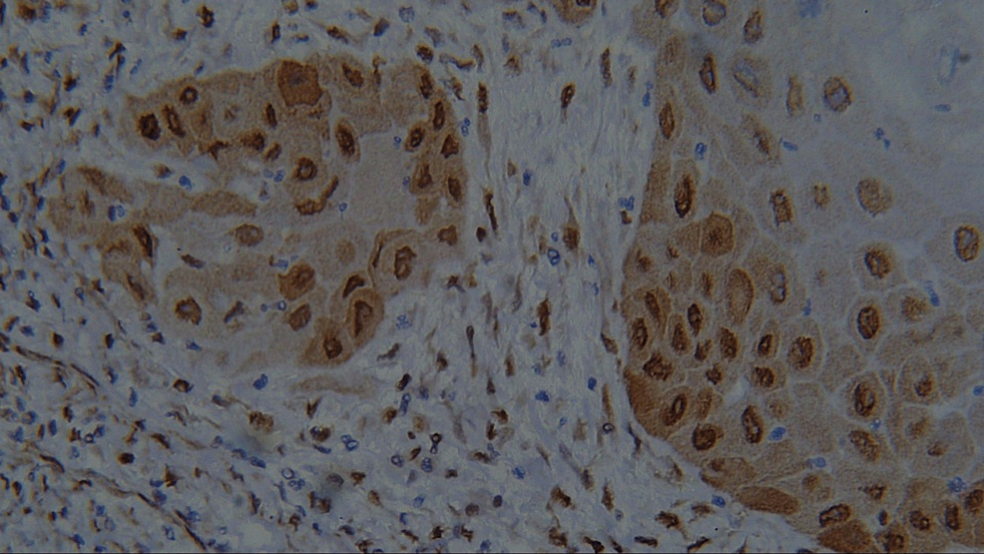
Photomicrograph showing expression of PCNA in WDSCC (IHC staining; 400x). IHC, immunohistochemistry; PCNA, proliferating cell nuclear antigen

**Figure 7:**
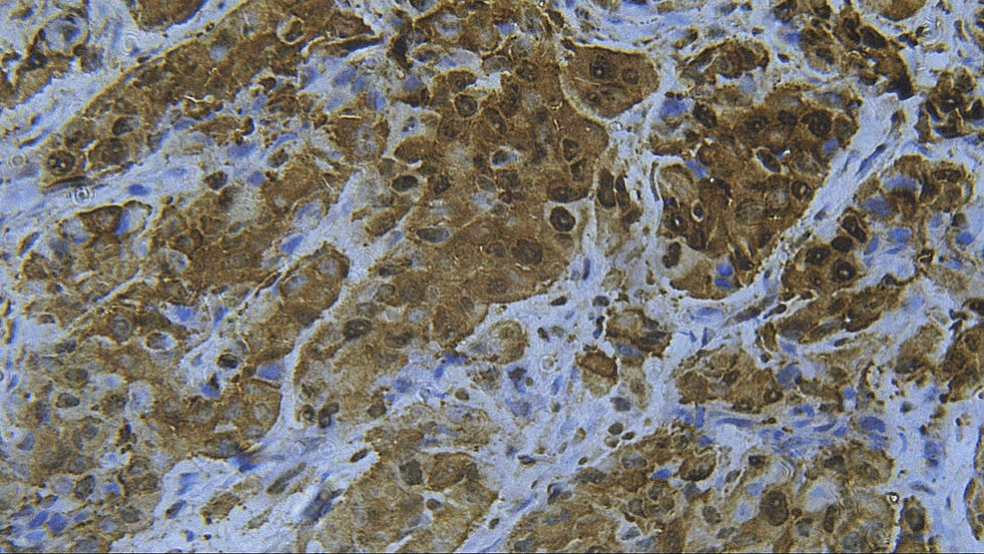
Photomicrograph showing expression of PCNA in MDSCC (IHC staining; 400x). IHC, immunohistochemistry; PCNA, proliferating cell nuclear antigen

**Figure 8:**
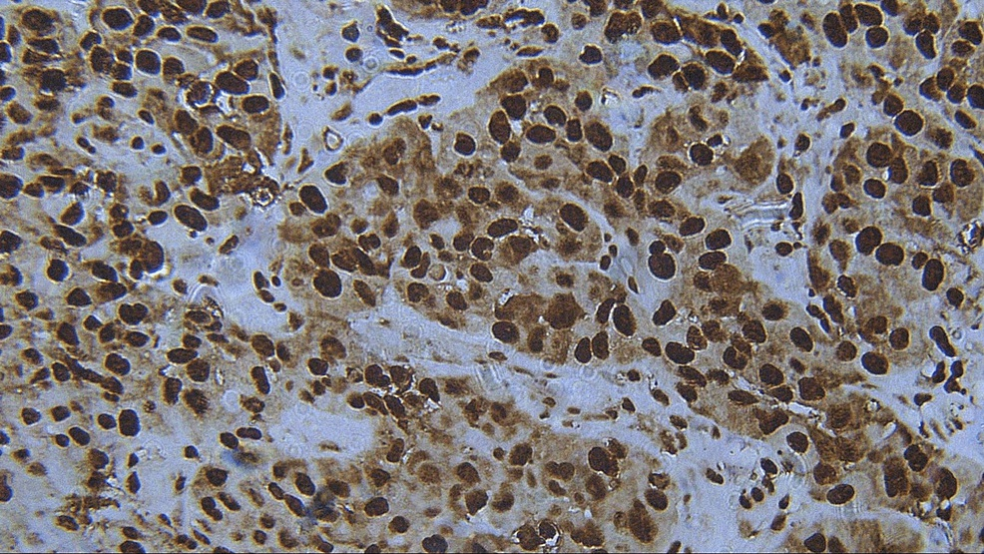
Photomicrograph showing expression of PCNA in PDSCC (IHC staining; 400x). IHC, immunohistochemistry; PCNA, proliferating cell nuclear antigen; PDSCC, poorly differentiated squamous cell carcinoma

**Figure 9:**
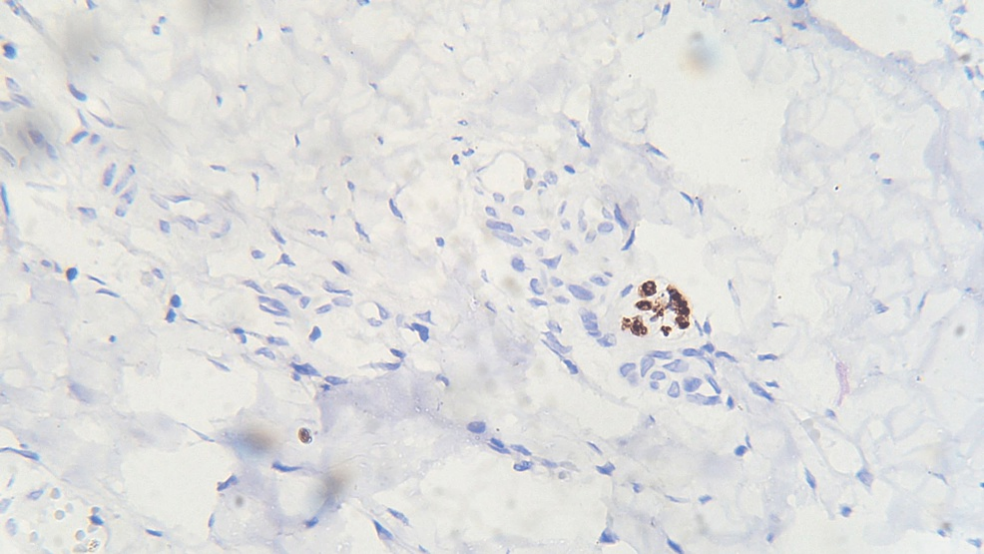
Photomicrograph showing expression of CD57 in normal mucosa (IHC staining; 400x). IHC, immunohistochemistry; CD57, cluster of differentiation 57

**Figure 10:**
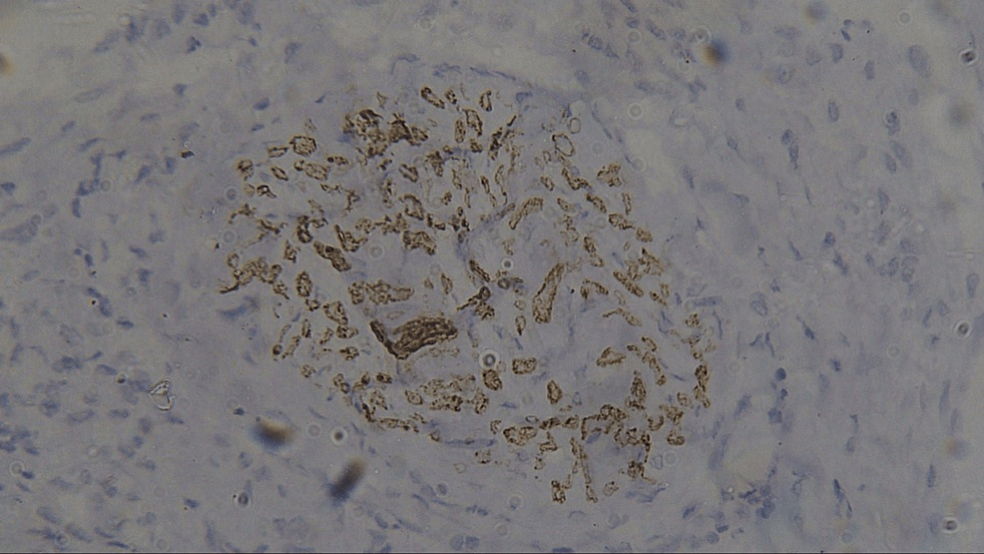
Photomicrograph showing expression of CD57 in WDSCC (IHC staining; 400x). IHC, immunohistochemistry; CD57, cluster of differentiation 57; WDSCC, well-differentiated squamous cell carcinoma

**Figure 11:**
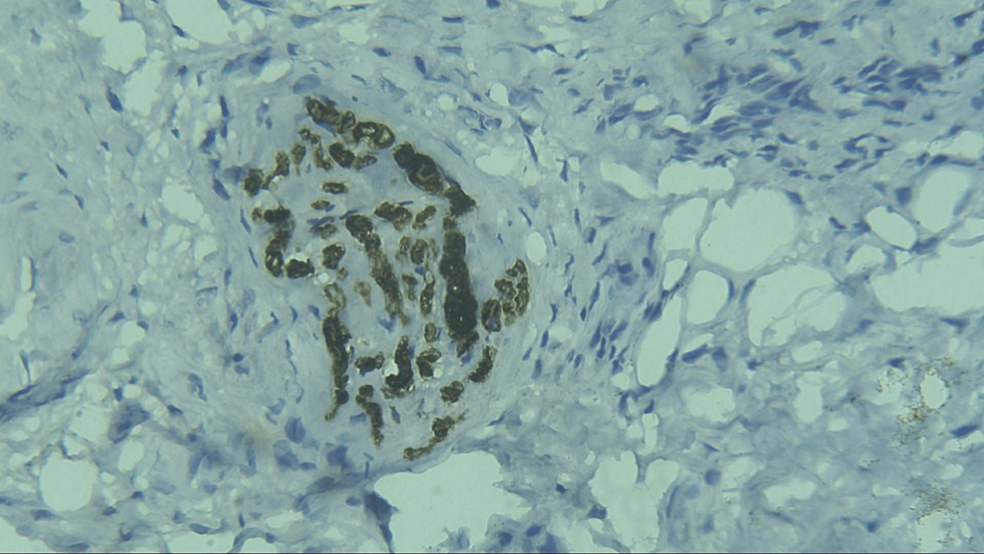
Photomicrograph showing expression of CD57 in MDSCC (IHC staining; 400x). IHC, immunohistochemistry; CD57, cluster of differentiation 57; MDSCC, moderately differentiated squamous cell carcinoma

**Figure 12:**
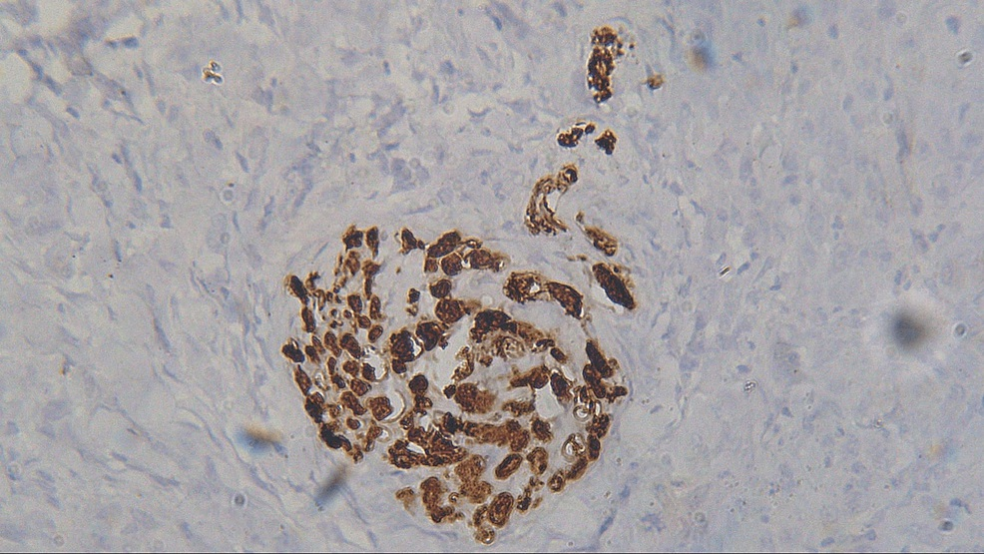
Photomicrograph showing expression of CD57 in PDSCC (IHC staining; 400x). IHC, immunohistochemistry; CD57, cluster of differentiation 57; PDSCC, poorly differentiated squamous cell carcinoma

Three sections were taken from each block and each section measured 4-5 micron thickness. One section was stained with the hematoxylin and eosin stain to reconfirm the diagnosis (Figures 1-4) while the other two sections were subjected to immunohistochemical staining for PCNA and CD57 respectively. The ribbons of tissue sections were transferred onto the Poly L-lysine coated slides. The slides were dewaxed by heating at 60°C for 60 min followed by deparaffinization in xylene for half an hour. The slides were then kept in absolute alcohol for 10 min followed by descending grades of alcohol (90%, 80%, and 70%) each for 10 min. The sections were then washed in distilled water for 5 min. The slides with the tissue sections were put in sodium citrate buffer solution (pH 9.0) and kept in a microwave oven for 10 min at high power and allowed to cool for 30 min for antigen retrieval. Slides were washed in distilled water for 5 min and dried completely. The slides are then immersed in a wash buffer for 5 min. The slides were treated with hydrogen peroxide for 10 min to block the endogenous peroxidase enzyme activity followed by washing in wash buffer for 5 min. The slides were then subjected to one drop of protein block and kept for 10 min blotted dry. The tissue sections were then covered with primary antibodies and incubated for 1 h. For PCNA expression, rabbit monoclonal PCNA antibody PC10 and for CD57 expression, mouse monoclonal - CD57 antibody was used. A drop of horseradish peroxide secondary antibody was added to the sections and incubated for 30 min. Freshly prepared chromogen substrate DAB (diaminobenzidine tetrahydrochloride) was added to the tissue sections and kept for 5 min. The slides were counterstained with Mayer’s hematoxylin and mounted with DPX (distrene polystyrene xylene). Cells are considered positive for PCNA staining when the nucleus of the highly mitotic cells stains up in light brown (Figures 5-8). Cells are considered positive for CD57 if the cells take up the intracytoplasmic brown staining (chromogenic color) (Figures 9-12).

Counting of cells was done by a single examiner twice in order to eliminate the intra-examiner variability. A total of 1,000 tumors from three different high-power fields were counted. The CD57 labeling index was calculated according to Stelin et al. (2009) [10].

CD57 labeling index = (total number of positively stained cells/1000) x 100

PCNA index was calculated by the criteria given by Poosarla et al. (2015) [11].

PCNA labeling index = (total number of positively stained cells/1000) x 100

The data obtained in this study were analyzed using the SPSS version 16.0 (statistical package for social science) software (IBM Corp., Armonk, NY). To compare and correlate the expression of CD57 and PCNA expression in different grades of OSCC ANOVA post hoc followed by Dunnet t-test and Pearson’s correlation coefficient test were used. p-value less than 0.05 (p<0.05) was considered as statically significant.

## Results

In this study, the age range for samples in the study groups was 39-72 years, 53-75 years, and 35-70 years with a mean age of 56.79 years, 61.4 years, and 55.7 years in well, moderate, and poorly differentiated squamous cell carcinomas respectively. There was a male predilection in the ratio of 4:1 and 3:2 in well and moderately differentiated cases whereas equal sex predilection in the ratio of 1:1 was noticed in poorly differentiated squamous cell carcinoma. Documentation of the associated deleterious habits and common site of the lesion showed tobacco and betel chewing as the most prevalent cause and buccal mucosa as the most favorable site in all the three histopathological grades of squamous cell carcinoma.

**Figure 13:**
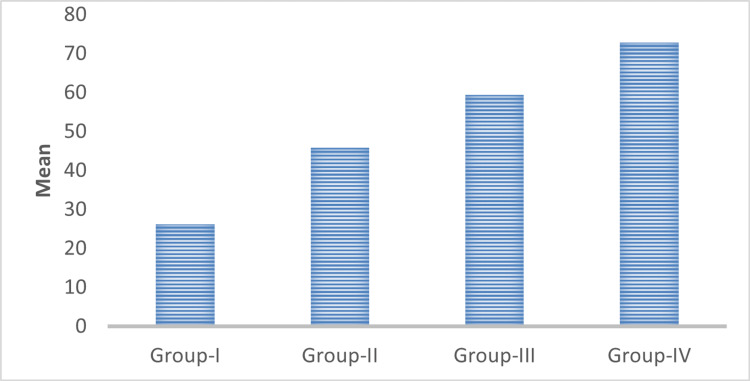
Comparison of the mean labeling index of PCNA between the groups. PCNA, proliferating cell nuclear antigen

The mean value of the PCNA labeling index was 26.19 ± 1.25, 45.88 ± 2.20, 59.38 ± 1.04, and 72.77 ± 4.35 in normal mucosa, well-differentiated OSCC, moderately differentiated OSCC, and poorly differentiated oral squamous cell carcinoma (OSCC) respectively (Table 1, Figure 13). Multiple comparison of the expression of the PCNA index between the WDSCC, MDSCC, and PDSCC study groups were statistically highly significant with a p-value of 0.001. Comparison of expression of the mean labeling index of PCNA between the study groups (WDSCC, MDSCC, and PDSCC) and the control group (normal mucosa) was also statistically highly significant with a p-value of 0.001.

**Table 1:** Mean PCNA labeling index in different groups. PCNA, proliferating cell nuclear antigen; WDSQCC, well-differentiated squamous cell carcinoma; MDSQCC, moderately differentiated squamous cell carcinoma; PDSQCC, poorly differentiated squamous cell carcinoma; SD, standard of deviation

Groups	Description	PCNA index (Mean ± SD)
Group I	Normal mucosa	26.19 ± 1.25
Group II	WDSQCC	45.88 ± 2.20
Group III	MDSQCC	59.38 ± 1.04
Group IV	PDSQCC	72.77 ± 4.35

**Figure 14:**
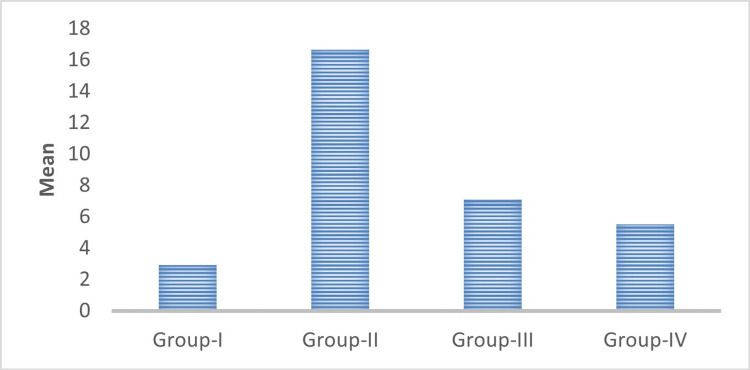
Comparison of the mean labeling index of CD57 between the groups. CD57, cluster of differentiation 57

Similarly, the mean value of the CD57 labeling index was 2.91 ± 0.82, 16.63 ± 2.33, 7.09 ± 1.41, and 5.53 ± 1.20 in normal mucosa, well-differentiated OSCC, moderately differentiated OSCC, and poorly differentiated OSCC respectively (Table 2, Figure 14). Multiple comparisons of expression of the CD57 labeling index between the WDSCC, MDSCC, and PDSCC study groups were statistically highly significant with a p-value of 0.001. Comparison of expression of the mean labeling index of CD57 between the WDSCC, MDSCC, and PDSCC study groups with that of the control group (normal mucosa) was also statistically highly significant with a p-value of 0.001. 

**Table 2:** Mean CD57 labeling index in different groups. CD57, cluster of differentiation 57; WDSQCC, well-differentiated squamous cell carcinoma; MDSQCC, moderately differentiated squamous cell carcinoma; PDSQCC, poorly differentiated squamous cell carcinoma; SD, standard deviation

Groups	Description	Labeling index (Mean ± SD)
Group I	Normal mucosa	2.91 ± 0.82
Group II	WDSQCC	16.63 ± 2.33
Group III	MDSQCC	7.09 ± 1.41
Group IV	PDSQCC	5.53 ± 1.20

**Figure 15:**
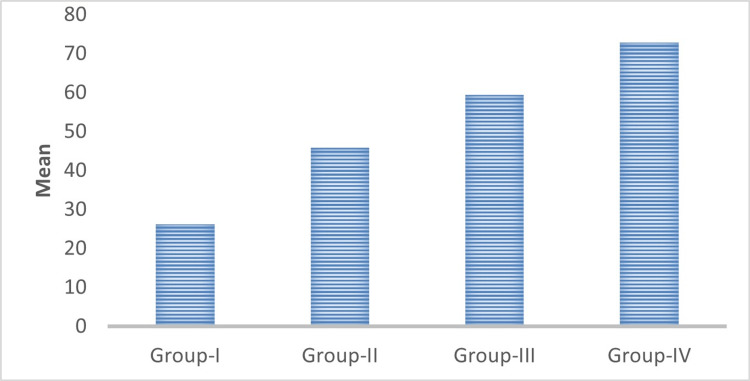
Correlation of the PCNA labeling index with the CD57 labeling index between the groups. PCNA, proliferating cell nuclear antigen; CD57, cluster of differentiation 57

In the present study on the correlation of the mean labeling index of PCNA and CD57, the Pearson correlation coefficient value within the groups was found not to be correlated. In contrast, the values were significant when compared between the groups (p<0.05) (Table 3, Figure 15).

**Table 3:** Correlation of the PCNA labeling index with the CD57 labeling index between the groups. PCNA, proliferating cell nuclear antigen; CD57, cluster of differentiation 57; WDSCC, well-differentiated squamous cell carcinoma; MDSCC, moderately differentiated squamous cell carcinoma; PDSCC, poorly differentiated squamous cell carcinoma *p<0.05 significant correlated between the groups

Correlation PCNA	CD57 labeling index (r value)				p Value
	Normal mucosa	WDSQCC	MDSQCC	PDSQCC	
Normal mucosa	-0.43	-0.67*	-0.78*	-0.89*	0.04
WDSQCC	-0.64*	-0.43	-0.76*	-0.92*	0.03
MDSQCC	-0.68*	-0.71*	-0.58	-0.96*	0.03
PDSQCC	-0.75*	-0.74*	0.81*	-0.59	0.03

## Discussion

Oral cancer is the most common malignancy, and it is the sixth most common cancer in the world [12]. The development of oral cancer is a multistep process that requires around four to seven events for the genetic changes to occur. These genetic alterations involve the classic hallmarks of malignancy and also undergo immortalization. As a result, the cells undergo behavioral and metabolic changes leading to uncontrolled proliferation [13].

Early diagnosis is vital as it reduces the severity and complications of the diseases, but it also helps in planning the treatment and prognosis. Recent advances in genomics and proteomics identify diseases by using gene or protein profiles. These advanced techniques use a multiple panel of markers which helps in the accurate identification of the diseased status of the individual. Recently, an immunohistochemical study also involved the use of multiple markers as an aid in the accurate diagnosis of diseases [10].

In the present study, both PCNA and CD57 were used, and the expression of these markers was analyzed in different grades of OSCC. After a thorough data search, this study is the first documented study where PCNA and CD57 were used as combined markers to find out the aggressiveness of the lesion and the immune status of the individual.

In this study, we evaluated the immunohistochemical expression of PCNA and CD57 in 10 cases each of well-differentiated, moderately differentiated, and poorly differentiated squamous cell carcinomas (Figures 5-12). The PCNA index was calculated according to the criteria given by Poosarla et al. (2015) [11]. The PCNA index was 26.19 ± 1.25, 45.88 ± 2.20, 59.38 ± 1.04, and 72.77 ± 4.35 for normal mucosa, WDSCC, MDSCC, and PDSCC respectively (Table 1, Figure 13). Higher expression of PCNA was found in poorly differentiated squamous cell carcinoma. The intensity of staining and the number of positive PCNA cells increased gradually from Group I to Group IV. Shin et al. (1993) analyzed the expression of PCNA in OSCC. In their study, the PCNA was increased 4-10-fold from adjacent normal epithelium to squamous cell carcinoma [14]. Girod et al. (1994) in their study found that there is a steady increase in PCNA expression from normal mucosa to moderately differentiated squamous cell carcinoma [15]. Zain et al. (1995) opined that PCNA is a good marker in differentiating normal epithelium and dysplastic epithelium [7].

Abdulkadir et al. (2010) from their study suggested that the expression of PCNA was a good indicator of the aggressiveness of the lesion [16]. Madan et al. (2015) showed that PCNA was a good marker in differentiating normal epithelium from oral squamous cell carcinoma [9]. Poorsala et al. (2015) showed that the proliferation of cells was increased from normal to premalignant lesions and to OSCC and they concluded that the PCNA marker was useful in predicting the aggressiveness of the lesion [11]. Keshav et al. (2015) studied the expression of PCNA in oral submucous fibrosis and concluded that PCNA was an indicator of the biological behavior of the lesion [17]. Ahmed et al. (2017) opined that the proliferation of cells was more in poorly differentiated followed by moderately differentiated squamous cell carcinoma and well-differentiated squamous cell carcinoma [18]. This study also showed a steady increase in PCNA expression with increasing grades of OSCC.

In the present study, the expression of the CD57 labeling index was 2.91 ± 0.82, 16.63 ± 2.33, 7.09 ± 1.41, and 5.53 ± 1.20 in normal mucosa, WDSCC, MDSCC, and PDSCC respectively (Table 2, Figure 14). It was found that the CD57 expression was increased from the normal mucosa to well-differentiated squamous cell carcinoma. According to Karpathiou et al. (2017), in head and neck tumors, there is a dense infiltration of cytotoxic T lymphocytes, NK cells, and dendritic cells. These cells are increased in number in order to kill the tumor cells [19]. Therefore, in well-differentiated OSCC the CD57 expression was increased. In moderately differentiated and poorly differentiated OSCC, the CD57 expression was decreased compared to well-differentiated OSCC but increased in comparison with the normal mucosa. This clearly indicates that rich infiltration of NK cells is associated with increased survival of the patient. The lack of these cells in moderately differentiated and poorly differentiated squamous cell carcinoma indicates a poor survival rate.

Fang et al. (2017) opined a high CD57 expression in the early stage of the disease and found that strong CD57 expression in OSCC could be an independent marker for longer survival [20]. Agarwal et al. (2016) opined that the CD57 mean labeling index was higher in alive patients (10.67) than in dead patients (3.67). This study concluded that a higher CD57 labeling index had a significant correlation with the status of the life [3]. Taghavi et al. (2015) indicated that high CD57 expression was associated with longer overall survival of the patient [21]. The present study also showed a high CD57 expression in well-differentiated squamous cell carcinoma and lower CD57 expression in moderately differentiated and poorly differentiated squamous cell carcinomas that are associated with decreased survival of the patient.

Iida et al. (2014) showed that increased CD57+T-cell infiltration in the tumor microenvironment was a potent prognostic marker for OSCC [22]. Zancope et al. (2010) showed that infiltration of NK cells (CD57) and CD 8+ cells in the tumor microenvironment reflected a favorable cytotoxic immune response against malignant cells [23]. These findings were in accordance with the present study interpreting that high CD57 expression in well-differentiated squamous cell carcinoma will have a better prognosis whereas low expression of CD57 in moderately differentiated followed by poorly differentiated squamous cell carcinoma has poor prognosis.

In the present study, the correlation between the PCNA and the CD57 labeling index within the groups is not significant. However, the correlation of PCNA and CD57 was found to be significant between the groups (Table 3, Figure 15). PCNA and CD57 are found to be good indicators of the aggressive nature of the lesion and the immune status of the patient respectively [3, 11]. The combination of PCNA and CD57 was found to be effective in identifying patients with good or poor survival rates and thereby it helps in planning the treatment modalities.

A highly significant increase in the PCNA labeling index was seen from normal mucosa to WDSCC, followed by MDSCC and PDSCC [3, 17]. Maximum proliferative index was noted in PDSCC [18]. Similarly, a highly significant decrease in the CD57 labeling index was seen from WDSCC followed by MDSCC and PDSCC. Maximum CD57 index was noted in WDSCC [19]. In correlation with the Pearson correlation coefficient test, PCNA and CD57 were found to be correlated between the groups. This clearly indicates that PCNA and CD57 are related to each other in different grades of OSCC.

Oral squamous cell carcinomas with higher PCNA expression had significantly shorter 5-year overall survival than those with lower PCNA expression. These findings implicated that high PCNA expression may have an impact on the 5-year overall survival of OSCC patients [24]. The poor overall prognosis and disease-free survival of OSCC were significantly predicted by high PCNA expression [25]. In clinical early-stage OSCC, high infiltrations of CD20+ B cells and CD57+ NK cells suggested a better overall survival rate. Notably, lower infiltrations of CD57+ NK cells and CD20+ B cells were independent predictors of poor OS in clinical early-stage OSCC. Thus, these markers are helpful in indicating the overall survival rate of OSCC patients [26].

Limitations

Our study only used a small number of samples; larger sample sizes will be needed in future research to accurately anticipate how PCNA and CD57 will be used in various grades of OSCC.

## Conclusions

It was concluded that as the grades of OSCC increase, the aggressive nature of the lesion increases, and the immune status of the patient decreases. Henceforth, the combination of PCNA and CD57 can be used as a valuable marker to detect the malignant potential of the lesion and to determine the survival rate of the patient wherein it helps in the stratification of patients for planning treatment modalities. Recognition and stratification of patients will provide a key factor for the success of treatment and early identification of the disease will also improve the survival rate of the patient.
